# MiR-499 Regulates Cell Proliferation and Apoptosis during Late-Stage Cardiac Differentiation via Sox6 and Cyclin D1

**DOI:** 10.1371/journal.pone.0074504

**Published:** 2013-09-11

**Authors:** Xianhui Li, Jiaji Wang, Zhuqing Jia, Qinghua Cui, Chenguang Zhang, Weiping Wang, Ping Chen, Kangtao Ma, Chunyan Zhou

**Affiliations:** 1 Department of Biochemistry and Molecular Biology, School of Basic Medical Sciences, Key Laboratory of Molecular Cardiovascular Sciences, Ministry of Education of China, Peking University, Beijing, China; 2 Department of Biomedical Informatics, School of Basic Medical Sciences, Peking University, Beijing, China; National Institutes of Health, United States of America

## Abstract

**Background:**

MiR-499 is a cardiac-abundant miRNA. However, the biological functions of miR-499 in differentiated cardiomyocytes or in the cardiomyocyte differentiation process is not very clear. Sox6 is believed to be one of its targets, and is also believed to play a role in cardiac differentiation. Therefore, our aim was to investigate the association between Sox6 and miR-499 during cardiac differentiation.

**Methodology/Principal Findings:**

Using a well-established *in* *vitro* cardiomyocyte differentiation system, mouse P19CL6 cells, we found that miR-499 was highly expressed in the late stage of cardiac differentiation. In cells stably transfected with miR-499 (P-499 cells), it was found that miR-499 could promote the differentiation into cardiomyocytes at the early stage of cardiac differentiation. Notably, cell viability assay, EdU incorporation assay, and cell cycle profile analysis all showed that the P-499 cells displayed the distinctive feature of hyperplastic growth. Further investigation confirmed that miR-499 could promote neonatal rat cardiomyocyte proliferation. MiR-499 knock-down enhanced apoptosis in the late differentiation stage in P19CL6 cells, but overexpression of miR-499 resulted in a decrease in the apoptosis rate. Sox6 was identified as a direct target of miR-499 and its expression was detected from day 8 or day 10 of cardiac differentiation of P19CL6 cells. Sox6 played a role in cell viability, inhibited cell proliferation and promoted cell apoptosis in P19CL6 cells and cardiomyocytes. The overexpression of Sox6 could reverse the proliferation and anti-apoptosis effects of miR-499. It was also found that miR-499 might exert its function by regulating cyclin D1 via its influence on Sox6.

**Conclusions/Significance:**

miR-499 probably regulates the proliferation and apoptosis of P19CL6 cells in the late stage of cardiac differentiation via its effects on Sox6 and cyclin D1.

## Introduction

Heart morphogenesis and development is a complicated process, in which cell cycle progression/exit control is of paramount importance. During the embryonic and fetal stages, cardiomyocytes rapidly proliferate so that a sufficient number of cells are produced to form the myocardium [1]. Before birth, proliferation ceases and cardiomyocytes throughout the myocardium undergo a hyperplastic to hypertrophic transition in which the predominant form of growth is an increase in cell size and myofibril density rather than cell number [2–4]. After birth, usually in the first two weeks of life in mice, neonatal cardiomyocytes complete terminal differentiation and the cell cycle is permanently arrested [5,6]. This phenomenon is common, but details of the mechanisms are currently not very clear.

Growing evidence indicates that microRNAs (miRNAs), which are endogenous regulatory RNAs, play important roles in heart development and heart pathogenesis. miR-499 is an miRNA that is abundantly found in cardiac cells and is essentially undetectable in human cardiac stem cells (hCSCs) or human embryonic stem cells (hESCs), but is expressed in differentiated or post-mitotic cardiomyocytes and continues to be expressed in fetal, neonatal, and adult cardiomyocytes [7–9]. However, the biological functions of miR-499 in differentiated cardiomyocytes or in cardiomyocyte differentiation is not very clear.

It is believed that one of the targets of miR-499 is Sox6, which is a member of the Sox transcription factor family and has been detected in a number of tissues [10,11]. There is evidence for the functionally diverse role of Sox6 in various cell types: it is involved in cartilage cell fate commitment [12], normal positioning and maturation of the cortical interneurons derived from medial ganglionic eminences [13], and erythroblast proliferation and normal erythrocyte maturation [14]. Sox6 is expressed in normal human and mouse heart [10,11]: in *p* ^100H^ mutant mice that lack a functional Sox6 gene, half of the homozygotes died within 24 h after birth and the remaining homozygotes that survived died within 2 weeks, which is believed to be caused by the disruption of the Sox6 gene [11]. In P19CL6 cells, an *in vitro* cardiomyocyte differentiation system, Sox6 expression was not detectable in the early stage of differentiation (before day 6); the highest expression was observed on day 11 and was associated with the initiation of cardiomyocyte beating [15]. This suggests that Sox6 is not involved in the fate commitment of cardiomyocytes (early stage of differentiation) but is associated with late-stage cardiomyocyte differentiation (terminal differentiation). However, details of the role of Sox6 in the process of heart development or cardiomyocyte differentiation are unclear.

Mouse Sox6-3’UTR has seven miR-499 target sites, three of which are conserved in its human, mouse, rat, dog and chicken counterparts. Numerous studies have demonstrated that miR-499 could target Sox6 via Sox6-3’UTR luciferase reporters [7,8,16,17]. During skeletal muscle atrophy, increased expression of Sox6 was associated with down-regulation of miR-499 [18]; in neonatal rat cardiomyocytes, Sox6 mRNA expression was significantly reduced after miR-499 overexpression [18,19]. How the association between miR-499 and Sox6 is related with the differentiation process of cardiomyocytes needs to be elucidated.

In this study, we investigated the impact of miR-499 and Sox6 during the differentiation process of cardiomyocytes by using the well-established *in vitro* cardiomyocyte differentiation system, P19CL6 cells. We found that Sox6 and miR-499 are highly expressed during cardiomyocyte terminal differentiation. By gain- and loss-of-function methods, including stable overexpression of miR-499 and Sox6 and transient down-regulation of miR-499 and Sox6, we demonstrate that Sox6, as a repressor of cyclin D1, arrests cardiomyocyte proliferation and facilitates cell cycle exit; miR-499 on the other hand downregulates the expression of its target protein, Sox6, to an appropriate level so as to prevent cardiomyocyte apoptosis.

## Materials and Methods

### Ethics statement

The animal experiments were carried out in strict accordance with the recommendations in the Guide for the Care and Use of Laboratory Animals of the National Institutes of Health. The protocol was consistence with by Kilkenny C et al [20] and approved by the Committee on the Ethics of Animal Experiments of the Peking University (LA 2010-066). All surgery was performed under sodium pentobarbital anesthesia, and all efforts were made to minimize suffering.

### Cell preparation and culture

Mouse P19CL6 cells [21,22], a cell line derived from P19 embryonal carcinoma cells [23], were kindly provided by Prof. Yunzeng. Zou, Fu Dan University, China. These cells can efficiently differentiate into beating cardiomyocytes with adherent conditions when exposed to 1% DMSO and thus P19CL6 is considered a useful *in vitro* model to study cardiomyocyte differentiation [21,22]. The cells were cultured as described previously [21]. In brief, the cells were grown in a 60-mm tissue culture dish with growth medium containing α-minimal essential medium (Gibco BRL) supplemented with 10% fetal bovine serum (FBS, Hyclone USA), penicillin (100 U/ml) and streptomycin (100 mg/ml), and were maintained in a 5% CO_2_ atmosphere at 37°C. To induce cardiac differentiation, P19CL6 cells were plated at a density of 3.7 × 10^5^ in a 60-mm tissue culture dish with the growth medium containing 1% dimethyl sulfoxide (DMSO). The medium was changed every 2 days. The days of differentiation were numbered consecutively beginning after the first day of the DMSO treatment (day 0).

Neonatal rat ventricular myocytes were prepared from 1- to 2-day-old Sprague-Dawley rats. Briefly, the rats were anesthetized with 5 mg/100 g body weight of sodium pentobarbital and heart organ was removed by classic surgery procedures. The ventricles were minced with scissors and digested at 37 ^o^C in phosphate-buffered saline (PBS) containing 0.1% trypsin (Gibco BRL) and 0.05% type I collagenase (Gibco BRL). After 8-10 cycles of digestion for 8 min each, the cells were collected by low speed centrifugation and suspended in DMED (Gibco BRL) with 15% fetal bovine serum (FBS, Gibco BRL). The resulting cell suspension, a mixture of myocytes and non-myocytes (mostly fibroblasts), was incubated in a tissue culture flask for 1.5 h for non-myocytes to adhere to the flask. The remaining unattached myocytes were appropriately diluted and plated in 60 mm dished (5×10^5^ cells/well). The cells were cultured in Dulbecco’s modified Eagle’s medium (DMEM, Gibco BRL) supplemented with 15% fetal bovine serum for 36 h before the transfection.

### Synthesis of pre-miR-499, anti-miR-499 and Sox6 siRNA oligonucleotides

Pre-miR-499, anti-miR-499, Sox6 siRNA and scrambled negative control were chemically synthesized by Genechem Co. Pre-miR-499 is single-stranded nucleotides containing two sequences that one is identical to mature miR-499, 5′-UUAAGACUUGCAGUGAUGUUU-3′, and another mimics the endogenous stem-loop. Anti-miR-499 is a 2′-*O*-methyl-modified single-stranded RNA: 5′-AAACAUCACUGCAAGUCUUAA-3′. The sequences for Sox6 siRNA are 5′-CACUUGUCAGUACCAUUCATT-3′ (sense) and 5′-UGAAUGGUACUGACAAGUGTT-3′ (antisense). In transient transfection, each oligonucleotide was transfected into cells using Lipofectamine 2000 (Invitrogen) at 50 nM according to the manufacturer’s recommendations.

### Expression plasmids construction and stable cell lines establishment

pcDNA3.1-Sox6 (containing the full-length cDNA of Sox6) was kindly provided by Dr. Veronique Lefebvre (Case Western Reserve University). To construct the miR-499 expression plasmid, a 453-bp DNA fragment encompassing pri-miR-499 was amplified by PCR from mouse genomic DNA, using the forward primer (*Xho*I site underlined) 5′-acacCTCGAGAGGTGAGGTCCAGACTGGGG-3′ and reverse primer (*Hind*III site underlined) 5′-gtacAAGCTTTGGTTAGGGAC CAGAGGGGA-3′. The amplified product (453 bp) was cloned into the pcDNA3.1(-) vector between the *Xho*I and *Hind*III restriction enzyme sites and verified by sequencing analysis. The plasmid was designated pcDNA3.1-miR-499. To create stable cell lines, 2 × 10^5^ P19CL6 cells were plated onto a 60-mm culture dish. When the cells reached approximately 50% confluence, P19CL6 cells were transfected with 2 µg pcDNA3.1-Sox6 plasmid, pcDNA3.1-miR-499 plasmid, or the control pcDNA3.1 plasmid using Lipofectamine 2000. G418 selection (600 µg/ml) was continued for 14–21 days, and viable clones were picked and expanded. Identification of P19CL6-miR-499 and P19CL6-Sox6 stable cell lines was performed by examination for expression of miR-499 and Sox6, respectively.

### Luciferase reporter construction and luciferase assays

The Sox6-3’-UTR luciferase reporter was prepared by amplifying the 720-bp DNA fragment of Sox6-3’UTR, which harbors three highly conserved predicted miR-499-binding sites. The forward primer (*Sac*I site underlined) 5′-tagaGAGCTCGACATTTCGCTCCCTTTCCC-3′ and reverse primer (*Hind*III site underlined) 5′-agagAAGCTTACTGTGGCAGCCTTGCTCAT-3′ were used for amplification of the reverse-transcribed cDNA of P19CL6 cells. The amplified product was cloned into the pMIR-REPORT luciferase plasmid (Ambion) between the *Sac*I and *Hind*III restriction enzyme sites to form the Sox6-3’-UTR pMIR-REPORT, and was verified by sequencing analysis. Mutant Sox6-3’UTR luciferase reporter was generated by PCR-based mutagenesis with the following primers: for the first binding site mutation, forward primer 5′-TGTCAAAGATTGTCTGAGACTTTGCA-3′ and reverse primer 5′-CAGACAATCTTTGACATCTTAAAATA-3′; for the second site, forward primer 5′-TGCAGTGTCTCCTGTGAGACTTTTAA-3′ and reverse primer 5′-CACAGGAGACACTGCAAAGTCTTAGA-3′; for the third site, forward primer 5′-GTAAGACAGAAATGTGAGACTCATAA-3′ and reverse primer 5′-CACATTTCTGACTTACTATTTTTCTT-3′. The mutated bases are underlined. All mutations were verified by sequencing analysis.

For luciferase analysis, HeLa cells were plated into 24-well plates at 5 × 10^4^ cells/well 24 h before transfection. pMIR-REPORT plasmid (200 ng) containing the Sox6-3’-UTR or its mutants and 20 ng control Renilla vector (phRLTK, Promega) were cotransfected with 2 µl transfection reagent (Roche) in triplicate. Pre-miR-499 duplex or scrambled negative control at a final concentration of 50 nM with Lipofectamine 2000 was added into each well. Lysates were collected 36 h after transfection, and luciferase activity was measured with the dual luciferase assay (Promega).

### Western blotting analysis

Total protein extracts were obtained with lysis buffer (150 mM NaCl, 10 mM Tris [pH 7.2], 5 mM EDTA, 0.1% sodium dodecyl sulfate [SDS], 1% sodium deoxycholate, 1% Triton X-100) containing protease inhibitor cocktail (Sigma). Proteins were separated by electrophoresis on 10–15% SDS-polyacrylamide gels, transferred to nitrocellulose, and incubated with primary antibodies. The rabbit polyclonal anti-Sox6 antibody (1:500), anti-cyclin D1 antibody (1:200) and anti-caspase 3 antibody (1:200) were from Abcam Ltd. The membrane was also probed for mouse glyceraldehyde-3-phosphate dehydrogenase (GAPDH) or anti-α-tubulin as a loading control. The blots were next incubated with peroxidase-conjugated mouse or rabbit IgG secondary antibodies and developed using the enhanced chemiluminescence kit (Amersham) and Hyperfilm ECL (Amersham).

### RT-PCR and quantitative real-time PCR

For mRNA analysis, total RNA was isolated with TRIzol reagent (Invitrogen), and 5 µg total RNA was reverse transcribed with random primers for cDNA synthesis in the presence of RNase inhibitor. The cDNA was used for PCR with specific primers. PCR products were subjected to electrophoresis on 2% agarose gels. To analyze miRNA, a mirVana qRT-PCR miRNA detection kit (Ambion Co.) and mirVana qRT-PCR primer set for miR-499 (Ambion Co.) were used according to the manufacturer’s directions. The relative expression levels were normalized to that of U6.

### Flow cytometry analysis of apoptosis and cell cycle

For apoptosis analysis, cultured cells were harvested by trypsinization and washed with PBS. Cells (1 × 10^6^) from each sample were processed with the Annexin V FITC/PI apoptosis detection kit (BD Biosciences) according to the manufacturer’s instructions. For cell cycle analysis, harvested cells were fixed in 75% ethanol on ice for 2–4 h and collected and resuspended in 600 µl PBS containing 0.1% RNase (Sigma) for RNA digestion for 1–2 h at 37°C. Finally, cells were stained with propidium iodide (50 µg/ml final concentration, Sigma) for 15 min in the dark. Apoptosis and cell cycle distribution of the cells were then analyzed using FACS Calibur (BD Biosciences).

### Cell viability assay

Cell viability was determined using the Dojindo Cell Counting Kit-8 (CCK-8, Dojindo Laboratories). Cells were seeded into a 96-well plate at a density of 1 × 10^4^ cells per well; after 24 or 48 h, CCK-8 solution (10 µl in each well containing 100 µl of medium) was added. Plates were incubated at 37°C for 4 h, and the absorbance at 450 nm was then measured. All experiments were done in triplicate and performed three times.

### EdU incorporation assay

For the EdU incorporation assays, cells were cultured in confocal dishes at a density of 1 × 10^5^ P19CL6 cells or 3 × 10^5^ neonatal rat cardiomyocytes per dish for 24 h at 37°C. Then, 50 µM of EdU was added to each dish and cells were cultured for an additional 2 h for P19CL6 cells or 24 h for neonatal rat cardiomyocytes at 37°C. The cells were fixed with 4% formaldehyde for 15 min at room temperature and treated with 0.5% Triton X-100 for 20 min at room temperature for permeabilization. After washing with PBS three times, 100 µl of 1× Apollo® reaction cocktail was added to each well and the cells were incubated for 30 min at room temperature. Then, the cells were stained with 100 µl of Hoechst 33342 for 30 min and visualized under a fluorescence microscope (Olympus Corporation, Tokyo, Japan). The EdU incorporation rate was expressed as the ratio of EdU-positive cells to total Hoechst 33342-positive cells (blue cells).

For immunofluorescence analysis, rat cardiomyocytes grown on confocal dishes were fixed with 4% paraformaldehyde in PBS (pH 7.4) for 15 min at 4°C and permeabilized with 0.1% Triton X-100 for 3 min at room temperature. The primary antibody used for immunofluorescence staining was monoclonal anti-α-actinin from Sigma (catalogue No. A7811). The secondary antibody was fluorescein-conjugated goat anti-mouse IgG (Zhongshan Co.).

### Statistical analysis

The data are displayed as mean ± standard deviation (SD). Comparisons were analyzed by Student’s *t*-test or ANOVA. Significance was analyzed using the SPSS10.0 software, and a *p*-value <0.05 was considered to indicate statistical significance.

## Results

### MiR-499 was highly expressed in the late stage of cardiac differentiation in P19CL6 cells

**Figure 1:**
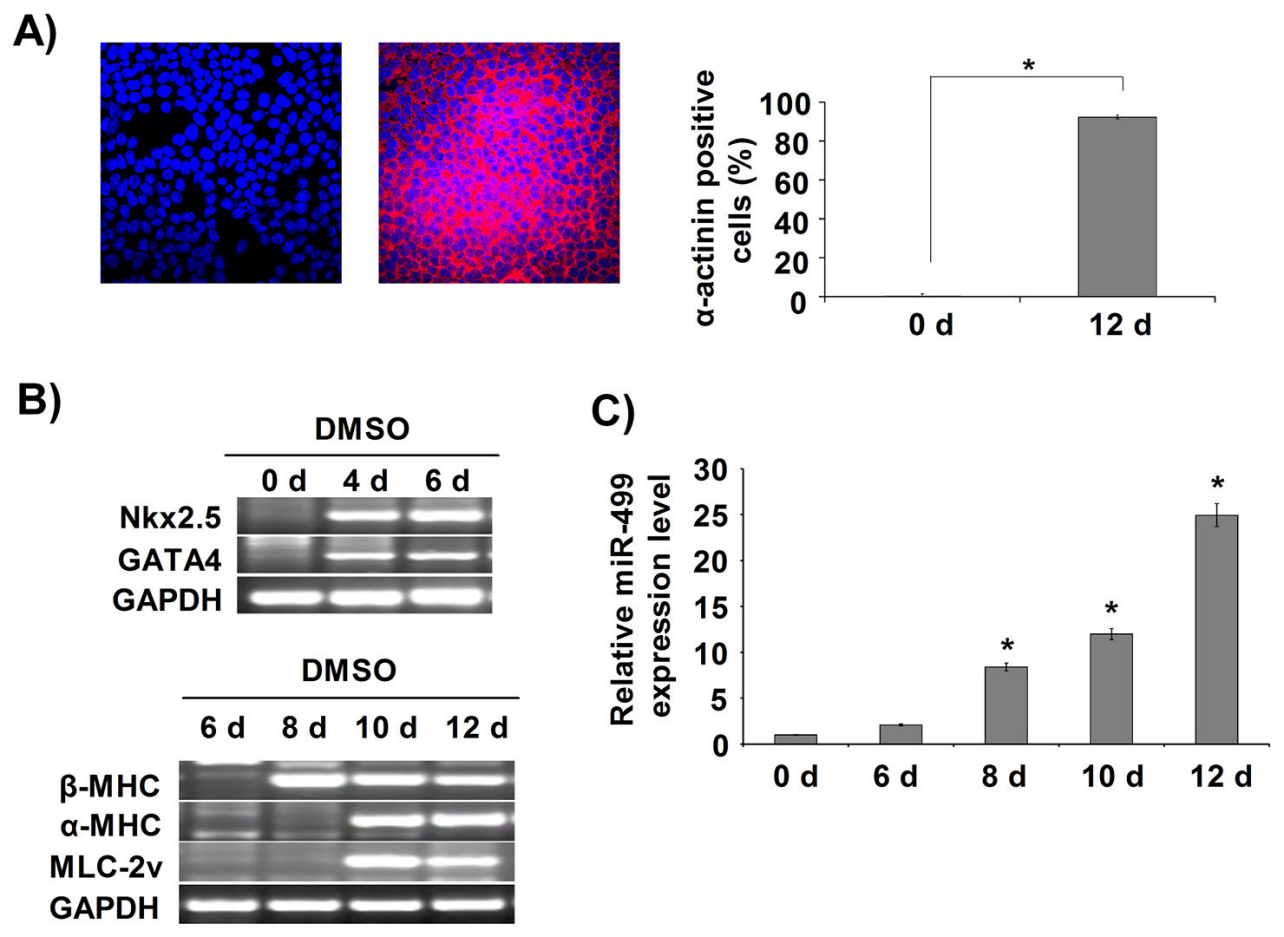
MiR-499 was highly expressed in the late stage of cardiac differentiation in P19CL6 cells. (**A**) P19CL6 cells were analyzed by immunofluorescence staining with a monoclonal antibody against sarcomeric α-actinin. Left, un-induced; right, DMSO-induced for 12 days. The positively stained cells were counted, and the percentage of positive cells is shown beside the images. (**B**) RT-PCR was performed to detect early cardiac-specific markers and cardiac contractile protein genes at the indicated time points. (**C**) qRT-PCR was performed to detect the expression of miR-499. U6 snRNA was used as an internal control. The results are given as relative value to the miR-499 expression level in P19CL6 cells at day 0 (0 d). Experiments were performed in triplicate and repeated three times; each bar represents mean ± S.D. * *P* < 0.05, vs. P19CL6 cells at 0 d.

When P19CL6 cells were treated with 1% dimethyl sulfoxide (DMSO) for 12 days, over 95% of the cells expressed sarcomeric α-actinin, a marker for mature cardiomyocytes, and rhythmic spontaneous beating started (Figure **1A**, Video **S1**). Cardiac-specific transcription factors GATA4 and Nkx2.5 were detected at day 4, an early stage of differentiation. Cardiac contractile proteins β-myosin heavy chain (β-MHC) and α-MHC were expressed at day 8 or day 10, the late stage of differentiation (Figure **1B**). To examine the temporal expression profile of miR-499 during cardiomyocyte differentiation, qRT-PCR for miR-499 was performed. The expression of miR-499 was almost undetectable at day 0 and day 6, but increased gradually from day 8, indicating that miR-499 might have some biological function in the late stage of cardiac differentiation of P19CL6 cells (Figure **1C**).

### MiR-499 promoted the proliferation of P19CL6 cells and neonatal rat cardiomyocytes

Given that miR-499 might participate in the cardiac differentiation of P19CL6 cells, we established a cell line that stably overexpressed miR-499 (P19CL6-miR-499, hereafter referred to as P-499) in order to observe the impact of persistent miR-499 expression on cardiac differentiation. P19CL6 cells stably transfected with pcDNA3.1 (P19CL6-pcDNA3.1, hereafter referred to as P-c3.1) was used as a negative control cell line. At the early differentiation stage, from day 0 to day 6, cell proliferation was similar among all three cell lines (Figure **S1A** **, a, b, c**). However, at the late differentiation stage, distinctive hyperplastic growth was observed in P-499 cells compared with P-c3.1 and P19CL6 cells (Figure **S1A, e, f, g**). As the cells formed a multilayer in the late differentiation stage, it was difficult to quantify the exact proliferation rate. To confirm this phenomenon observed in multilayer cell cultures, we employed the replating strategy. Using this method, the multilayer cells in the late differentiation stage could be observed in monolayer conditions. We replated the cells as a monolayer at the same density on day 10 of differentiation. After another 48 h of culture, the state of cell growth was observed under a microscope and the results confirmed our observations in multilayer cell cultures (Figure **S1B** **, a, b, c**).

**Figure 2:**
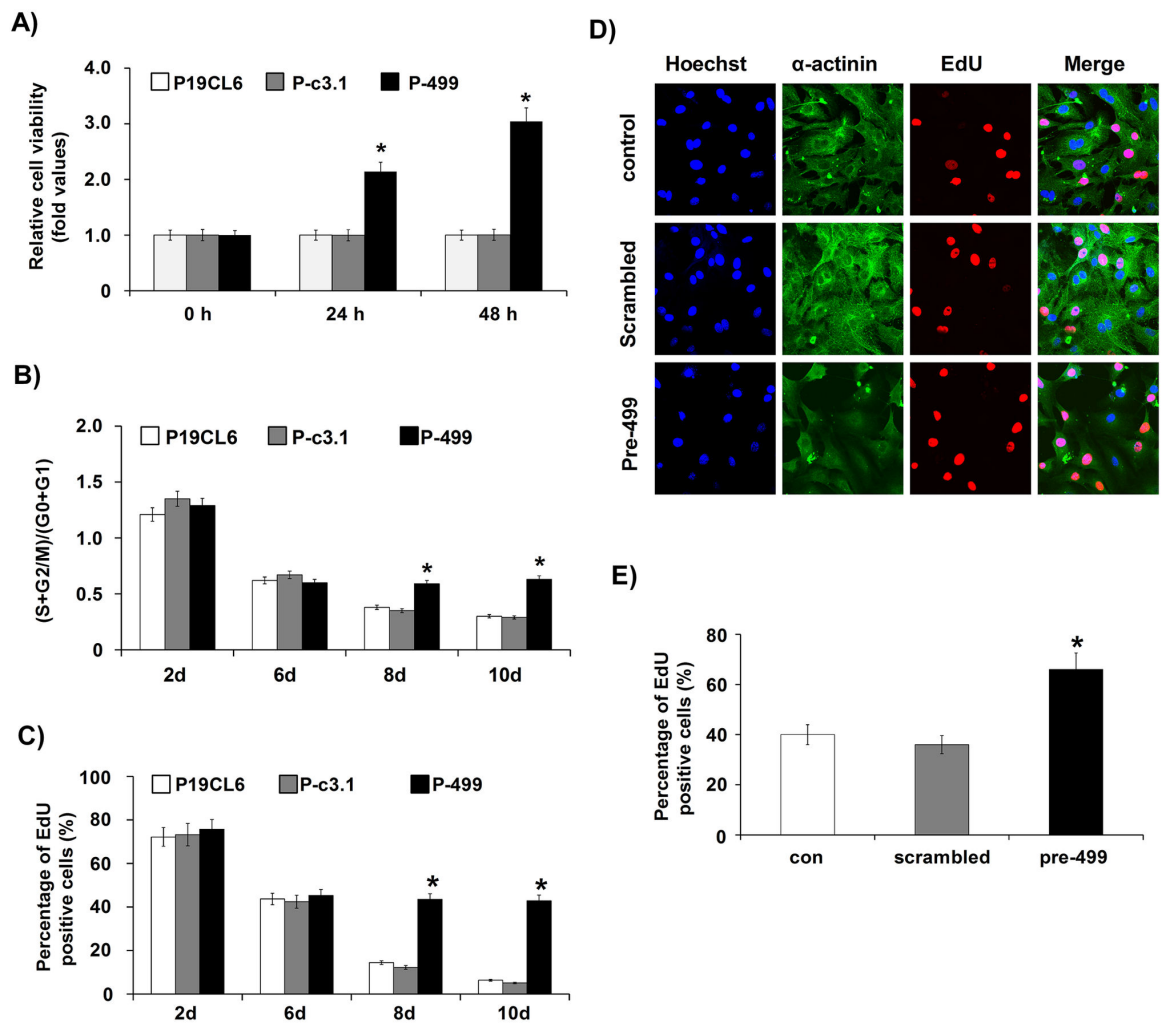
MiR-499 promoted the proliferation of P19CL6 cells and neonatal rat cardiomyocytes. (**A**) Cell viability assay was performed using CCK-8 on cells replated at day 10 of differentiation induction and cultured for 0, 24 and 48 h after replating. Experiments were performed in triplicate and repeated three times; each bar represents mean ± S.D. * *P* < 0.05, vs. P-c3.1cells. (**B**) Cell cycle analysis was performed by flow cytometry. The percentage of cells in each phase and the corresponding (S+G2/M)/(G0+G1) value was calculated for different cell lines at the same time points. Experiments were performed in triplicate and repeated three times; each bar represents mean ± S.D. * *P* < 0.05, vs. P-c3.1 cells. (**C**) EdU incorporation assay was performed. The percentage of EdU-positive cells was calculated for different cell lines at the same time points. Experiments were performed in triplicate and repeated three times; each bar represents mean ± S.D. * *P* < 0.05, vs. P-c3.1 cells. (**D**, **E**) EdU incorporation assay was performed in neonatal rat cardiomyocytes. Representative images are shown in Figure **2D**. The percentage of EdU-positive cells was compared among different cell groups, as shown in Figure **2E**. Experiments were performed in triplicate and repeated three times; each bar represents mean ± S.D. * *P* < 0.05, the cells transfected with pre-499 oligonucleotides vs. the cells transfected with scrambled oligonucleotides. More than 300 P19CL6 cells (**C**) or 200 neonatal rat cardiomyocytes (**D**, **E**), respectively, were counted in each condition for EdU assays. P-c3.1, P19CL6 cells stably transfected with pcDNA3.1 plasmid; P-499, P19CL6 cells stably transfected with pcDNA3.1-miR-499 recombinant plasmid.

Cell viability was analyzed using the CCK-8 assay at 0, 24 and 48 h after replating. The CCK-8 assay results showed that the cell viability of P-499 cells was 2.14- (24 h) and 3.04-fold (48 h) of that of P-c3.1 cells and P19CL6 cells (Figure **2A**), as also evidenced by the overgrowth observed under a microscope. Therefore, miR-499 overexpression promotes cell proliferation.

To understand the distinct proliferation phenotype of P-499 cells, we performed cell cycle profile analysis and EdU incorporation assay. The flow cytometry analysis indicated that miR-499 had significant effects on cell proliferation in the late stage of differentiation but not in the early stage. Similar percentages of cells in the G1 or S and G2 phases were observed at day 2 and day 6 in all three cell lines. However, by day 8 and day 10, the cell cycle progressed differently. In P19CL6 and P-c3.1 cells, the percentage of cells in the G1 phase increased from 72.27% and 74.30% respectively at day 8, to 76.76% and 77.31% respectively at day 10, indicating that the cells had almost stopped proliferating and might have started undergoing terminal differentiation; on the other hand, in P-499 cells, the percentage of cells in the G1 phase on day 8 (63.09%) and day 10 (61.5%) were at the same level as on day 6, which suggests that overexpression of miR-499 may maintain cell proliferation and thus inhibit terminal differentiation (Figure **2B**, **S2A**).

Consistent with the cell cycle analysis, the results of EdU incorporation were not significantly different among P19CL6, P-c3.1 and P-499 cells at the early stage of cardiac differentiation (day 2 and day 6): cells from all three cell lines possessed high proliferation capacity. However, at the late stage of cardiac differentiation, the incorporation of EdU gradually decreased in P19CL6 and P-c3.1 cells, but it was higher at day 8 and day 10 in P-499 cells (43.47% vs. 14.38% [P19CL6] and 12.21% [P-c3.1] on day 8; 42.85% vs. 6.32% [P19CL6] and 5.08% [P-c3.1] on day 10) (Figure **2C** **, S2B**); therefore, DNA synthesis gradually ceased and P19CL6 and P-c3.1 cells exited the cell cycle, while P-499 cells showed continuous proliferation.

To further confirm that miR-499 could promote cardiomyocyte proliferation during terminal differentiation, we tested whether miR-499 could promote proliferation of neonatal rat cardiomyocytes. We selected 1- or 2-day-old neonatal rats for preparing ventricular myocytes, which are known to undergo terminal differentiation. The ventricular cardiomyocytes were transfected with pre-miR-499 or scrambled oligonucleotides. EdU incorporation assay showed that EdU incorporation was almost 1.5-fold of that in pre-miR-499-transfected cardiomyocytes compared with cells that were not transfected or those that were transfected with scrambled oligonucleotides (Figure **2D, 2E**).

### MiR-499 knock-down enhanced apoptosis of cells in the late differentiation stage

**Figure 3:**
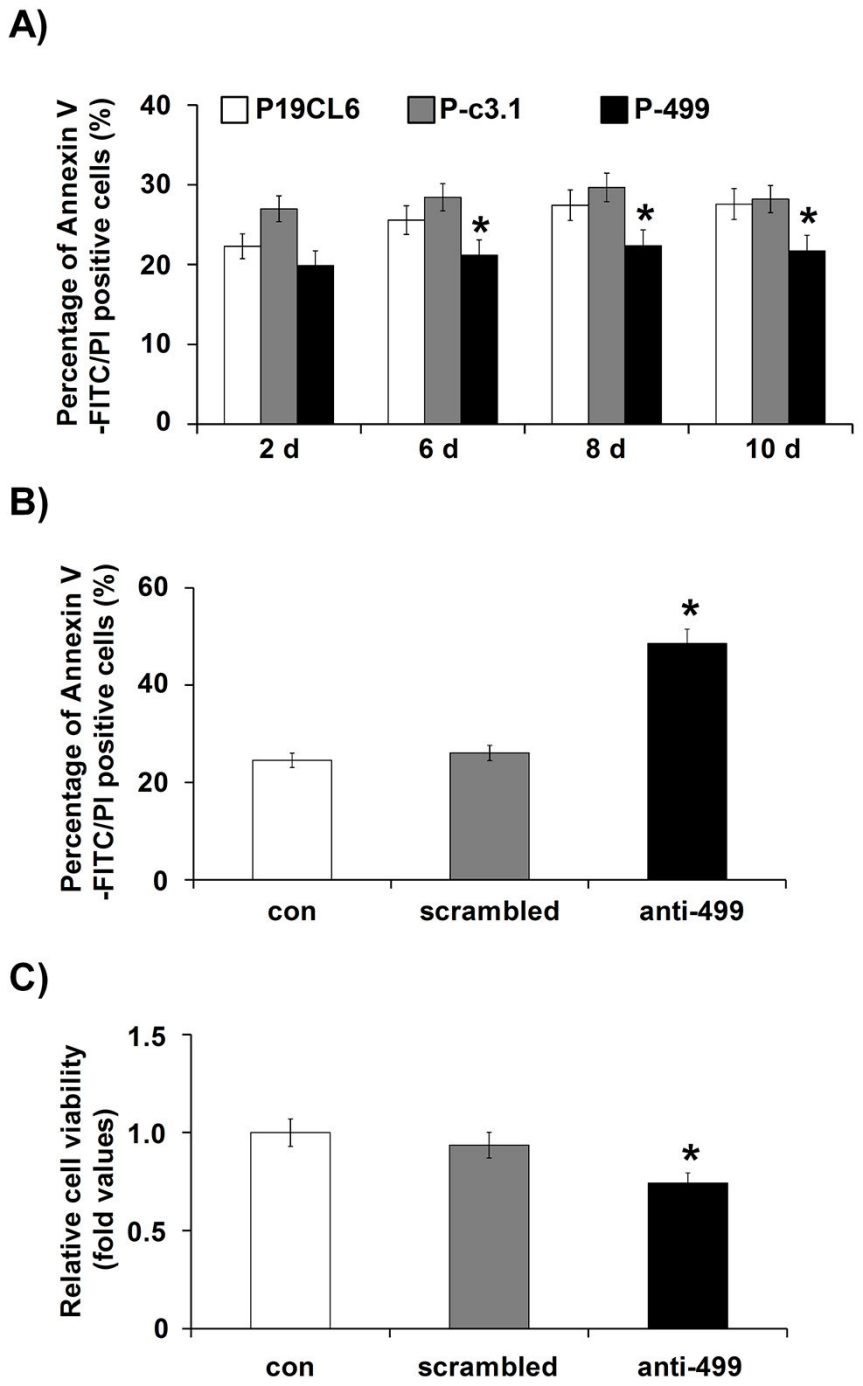
MiR-499 knock-down enhanced apoptosis of cells in the late differentiation stage. (**A**) Cell apoptosis was analyzed with annexin V-FITC and PI staining by flow cytometry at the indicated time. The apoptosis rate in different cell lines was calculated and compared at the same time points. Experiments were performed in triplicate and repeated three times; each bar represents mean ± S.D. * *P* < 0.05, vs. P-c3.1 cells. (**B**, **C**) P19CL6 cells were replated and transfected with anti-499 (anti-499) or scrambled oligonucleotides on differentiation day 12. After 48 h of transfection, the effects of anti-499 on apoptosis were examined using the annexin V-FITC binding assay (**B**) or CCK-8 assay (**C**). Experiments were performed in triplicate and repeated 3 times; each bar represents mean ± S.D. * *P* < 0.05, vs. the cells transfected with scrambled oligonucleotides. P-c3.1, P19CL6 cells stably transfected with pcDNA3.1 plasmid; P-499, P19CL6 cells stably transfected with pcDNA3.1-miR-499 recombinant plasmid.

Using the annexin V-FITC binding assay, we found that miR-499 overexpression resulted in a decreased apoptosis rate in P-499 cells compared with P19CL6 and P-c3.1 cells from day 6 to day 12 (Figure **3A** **, S2C**). To investigate the effects of endogenous miR-499 knock-down, we replated P19CL6 cells and transfected them with cholesterol-modified anti-miR-499 (anti-499) oligonucleotides at day 12 of differentiation. The cells that were not transfected or transfected with scrambled oligonucleotides were used as negative controls. After transfection with anti-499 for 48 h, the cells became round and detached from the plate in some areas. In contrast, the control cells maintained their fibroblast-like shape and adhered to the plate (Figure **S1C**). Therefore, miR-499 knock-down increased the apoptosis rate of cells at the late differentiation stage. The annexin V-FITC binding assay showed that silencing of miR-499 promoted cell apoptosis (Figure **3B** **, S2D**). A CCK-8 assay also indicated that transfection with anti-499 decreased cell viability (Figure **3C**).

### MiR-499 targeted Sox6 at the late stage of cardiac differentiation

**Figure 4:**
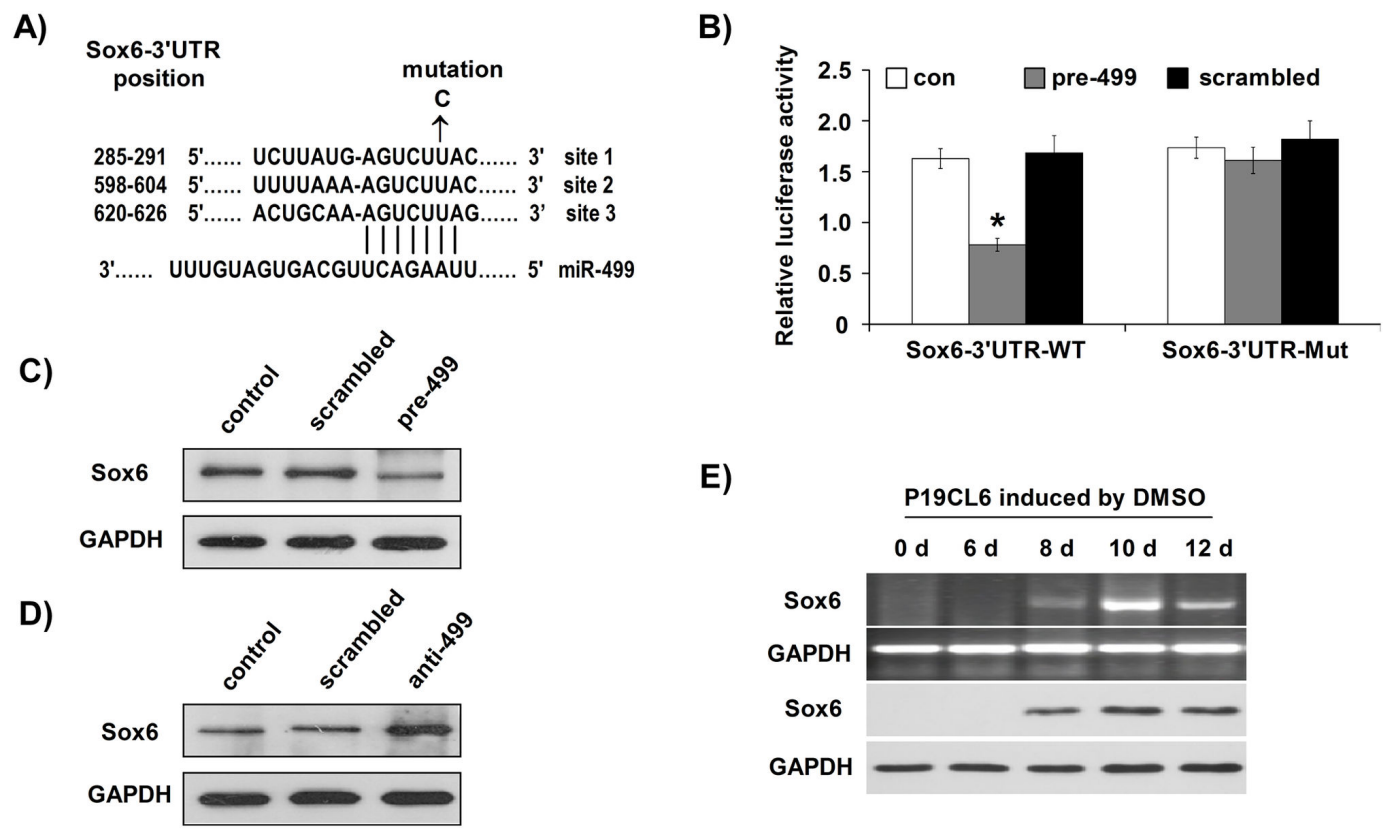
MiR-499 targeted Sox6 at the late stage of cardiac differentiation. (**A**) A schematic diagram showing the three highly conserved predicted miR-499-binding sites of Sox6-3’UTR. Nucleotide mutations are indicated by an arrow head. (**B**) Luciferase reporter assays were performed by co-transfection of pre-miR-499 oligonucleotides with a wild type Sox6-3’UTR luciferase reporter construct containing miR-499 binding sites (Sox6-3’UTR-WT) or mutant Sox6-3’UTR luciferase reporter construct containing mutated miR-499 binding sites (Sox6-3’UTR-Mut). (**C**, **D**) P19Cl6 cells transfected with pre-miR-499 oligonucleotides (**C**) or anti-499 oligonucleotides (**D**) were harvested and total protein was isolated for Western blotting analysis of endogenous Sox6 protein level. Scrambled oligonucleotides were used as the negative control. (**E**) RT-PCR and Western blotting were performed to determine endogenous expression of Sox6. GAPDH was used as an internal control.

Luciferase assay showed that addition of the pre-miR-499 duplex at a concentration of 50 nM resulted in decreased luciferase activity of the reporter that harbored Sox6-3’UTR (with the three highly conserved predicted miR-499-binding sites, Sox6-3’UTR-WT) in HeLa cells, but this was not observed with the reporter containing mutated miR-499-binding sites (Sox6-3’UTR-Mut) (Figure **4A, 4B**). The inhibitory effect of miR-499 on Sox6 translation might also be observed in cardiac differentiation of P19CL6 cells. Therefore, we replated P19CL6 cells at day 8 of differentiation, for pre-miR-499 transfection. After 48 h of pre-miR-499 transfection, Western blotting analysis showed that the Sox6 protein level was reduced dramatically (Figure **4C**). Next, we tested whether knock-down of endogenous miR-499 could influence endogenous Sox6 expression. As the maximum level of miR-499 expression was observed on day 12 of differentiation (Figure **1C**), P19CL6 cells were replated at day 12 of differentiation for anti-499 transfection. After 48 h of anti-499 transfection, Sox6 protein level was upregulated compared to the control groups (Figure **4D**), suggesting that the presumed repression of Sox6 by endogenous miR-499 could be attenuated by exogenous anti-499. The expression profile of Sox6 indicated that Sox6 was not detected until day 8 of induction, and that its expression reached the highest value on day 10 (Figure **4E**), by which time miR-499 expression started to increase (Figure **1C**). These results promoted us to investigate whether Sox6 is the target of miR-499 during cardiac differentiation of P19CL6 cells.

### Sox6 participated in cell proliferation, particularly in the apoptosis phase

We established a cell line that stably overexpressed Sox6 (P19CL6-Sox6, P-Sox6) in order to determine the function of Sox6. The P-c3.1 cell line was used as the negative control.

**Figure 5:**
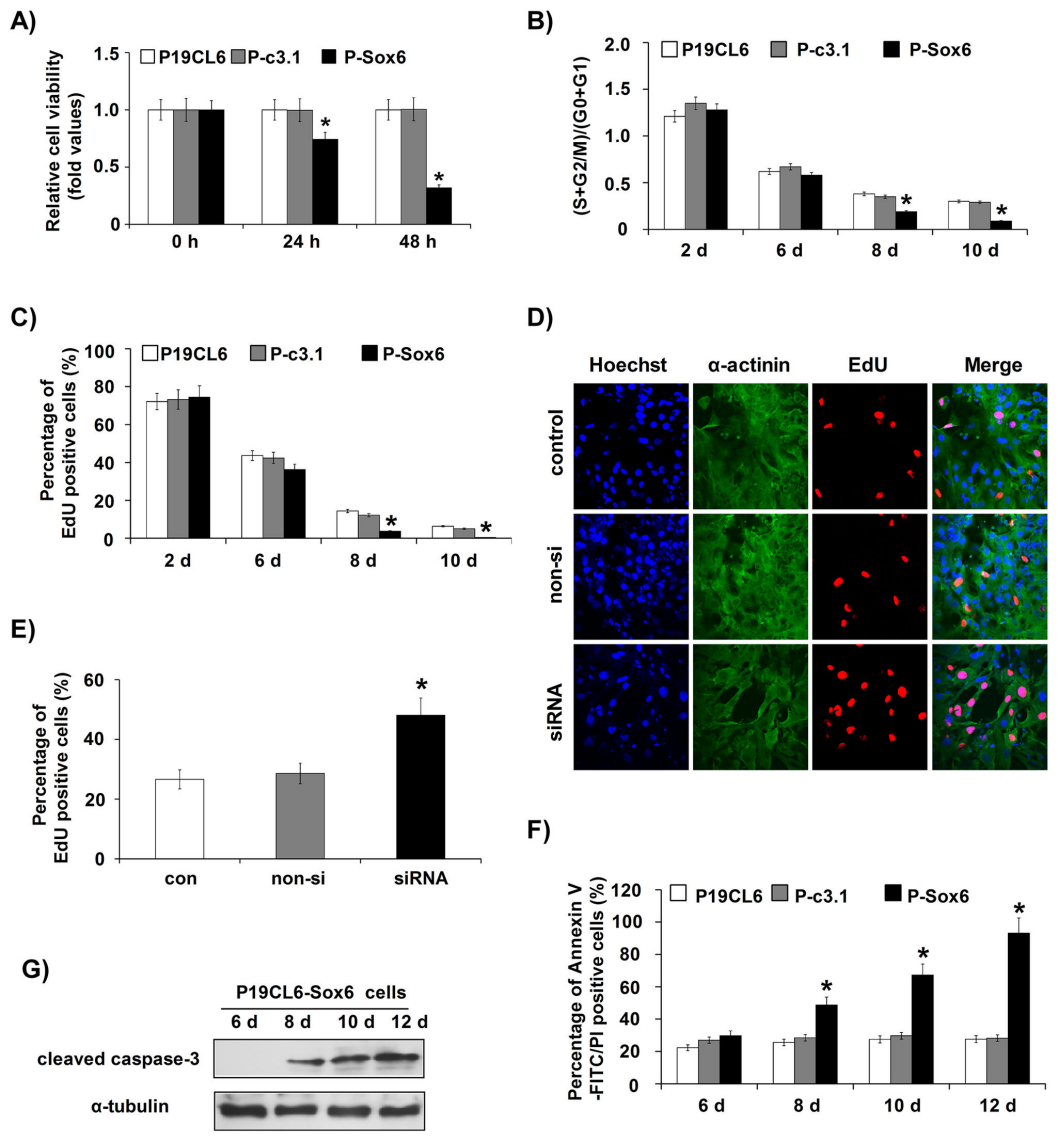
Sox6 participated in cell proliferation, particularly in the apoptosis phase. (**A**) Cell viability assay with CCK-8 was performed on cells replated at day 10 of induction and cultured for 0, 24 and 48 h after replating. Experiments were performed in triplicate and repeated three times; each bar represents mean ± S.D. * *P* < 0.05, vs. P-c3.1 cells. (**B**) Cell cycle analysis was performed by flow cytometry. The percentage of cells in each phase was calculated, and the (S+G2/M)/(G0+G1) value was determined for different cell lines at the same time points of cardiac differentiation. Each bar represents mean ± S.D. * *P* < 0.05, vs. P-c3.1 cells. (**C**, **D**) EdU incorporation assay of P19CL6 cells. The percentage of EdU-positive cells among different cell lines at the same time points was compared. More than 300 P19CL6 cells were counted for each condition. The experiments were repeated 3 times; each bar represents mean ± S.D. * *P* < 0.05, vs. P-c3.1 cells. (**D**, **E**) EdU incorporation assay. Representative images are shown in Figure **5D**. More than 200 neonatal rat cardiomyocytes were counted for each condition. The percentage of EdU-positive cells in cardiomyocytes treated with non-siRNA and siRNA was compared; each bar represents mean ± S.D. * *P* < 0.05, vs. non-si. (**F**) Cell apoptosis was analyzed with annexin V-FITC and PI staining by flow cytometry at the indicated times. The apoptosis rate was compared between different cell lines at the same time points. Experiments were performed in triplicate and repeated three times; each bar represents mean ± S.D. * *P* < 0.05, vs. P-c3.1 cells. (**G**) Cleaved caspase-3 in P-Sox6 cells at differentiation days 6, 8, 10 and 12 was detected using special anti-cleaved caspase 3 antibody by Western blotting analysis and α-tubulin was used as a control.P-c3.1, P19CL6 cells stably transfected with pcDNA3.1 plasmid; P-Sox6, P19CL6 cells stably transfected with pcDNA3.1-Sox6 recombinant plasmid.

Using the method mentioned before, the multilayer differentiating cells in the late differentiation stage were replated on day 10 of differentiation. P19CL6, P-c3.1, and P-Sox6 cells were all replated at the same original density. Observation of the cell monolayer showed that Sox6 overexpression blocked the proliferation of P19CL6 cells (Figure **S1A** **, a, b, d and e, f, h**). To verify this result, a cell viability assay using CCK-8 was performed at 0, 24 and 48 h. The results showed that the cell viability of P-Sox6 cells was 25% (24 h) and 70% (48 h) lower than that of P-c3.1 cells and P19CL6 cells (Figure **5A**).

Considering that P-Sox6 cells displayed decreased cell viability compared with the other cell lines, further investigation was carried out to explore whether Sox6 expression level is correlated with cell proliferation. Flow cytometric analysis demonstrated that compared to the P19CL6 and P-c3.1 cells, a greater proportion of P-Sox6 cells were in the G1 phase, especially at day 8 (83.82% vs. 72.27% [P19CL6] and 74.36% [P-c3.1]) and day 10 (91.76% vs. 76.76% [P19CL6] and 77.31% [P-c3.1]) (Figure **5B** **, S3A**). The EdU incorporation assay showed that EdU incorporation in P-Sox6 cells was lower compared with P19CL6 and P-c3.1 cells at day 8 and day 10 (day 8: 3.76% vs. 14.38% [P19CL6] and 12.21% [P-c3.1]; day 10: 0.43% vs. 6.32% [P16CL6] and 5.08% [P-c3.1]) (Figure **5C** **, S3B**). These results indicate that overexpression of Sox6 inhibited continuous proliferation.

We also tested whether Sox6 could inhibit the proliferation of neonatal rat cardiomyocytes. Cardiomyocytes were transfected with Sox6-specific RNAi nucleotides (siRNA) or non-silenced RNA nucleotides (non-siRNA). EdU incorporation assay showed that the EdU incorporation rate was higher in the siRNA group compared with the non-siRNA group (Figure **5D, 5E**).

Since Sox6 overexpression caused the cell numbers to gradually decrease after 8 days of differentiation, we supposed that overexpression of Sox6 may induce cell apoptosis. Therefore, we performed an annexin V-FITC binding assay and cleaved caspase-3 detection. The number of annexinV-FITC-positive P-Sox6 cells showed a marked increase from day 8 to day 12 compared with other groups at the same time points (Figure **5F** **, S3C**). Activation of caspase-3 was also noted from day 8 to day 12 in P-Sox6 cells. Cleaved caspase-3 was not detected in P-Sox6 cells on day 6 (Figure **5G**) or in other cells at any time point (data not shown).

### Sox6 reversed the proliferation and anti-apoptosis effects of miR-499

**Figure 6:**
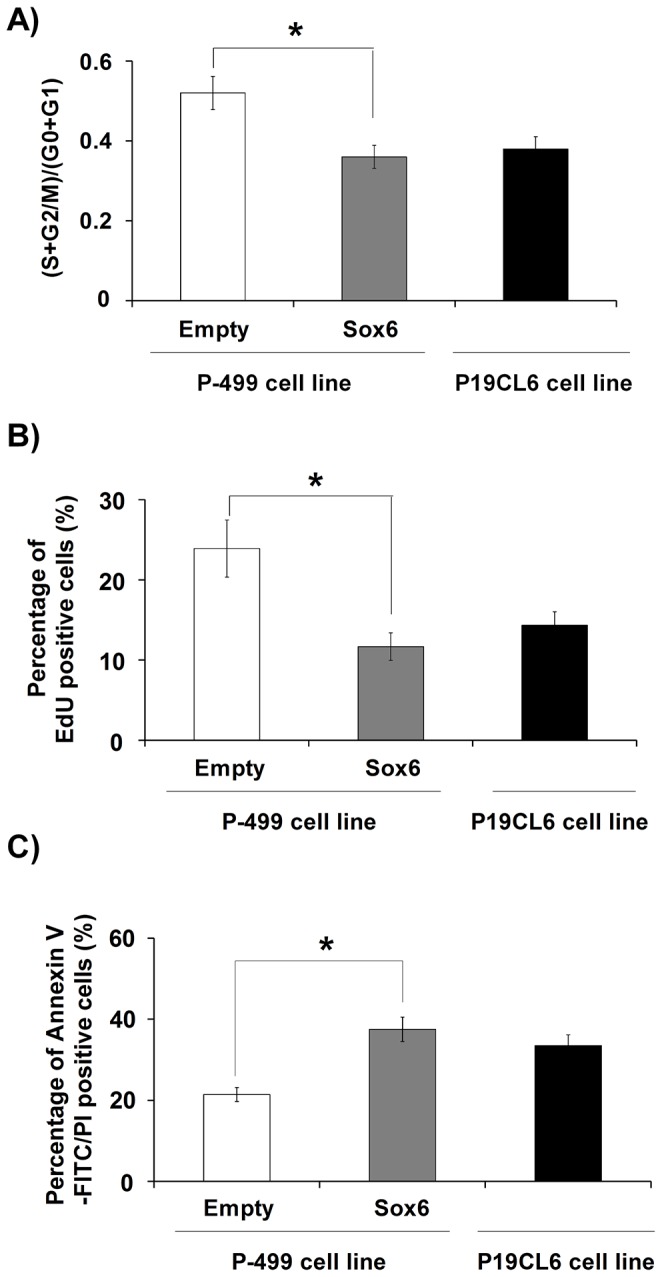
Sox6 reversed the proliferation and anti-apoptosis effects of miR-499. (**A**) Cell cycle analysis was performed by flow cytometry. At day 8 of differentiation, the (S+G2/M)/(G0+G1) value for the different cell lines was compared. The experiment was repeated three times. Each bar represents mean ± S.D. * *P* < 0.05, vs. empty or non-siRNA. (**B**) EdU incorporation assay was performed at day 8 of differentiation. The percentage of EdU-positive cells among different cell lines was compared. More than 300 P19CL6 cells were counted in each condition. The experiment was repeated three times. Each bar represents mean ± S.D. * *P* < 0.05, vs. empty or non-siRNA. (**C**) Cell apoptosis was analyzed with annexin V-FITC and PI staining by flow cytometry at day 8 of differentiation. The experiment was repeated three times. The apoptosis rate was calculated and compared. Each bar represents mean ± S.D. * *P* < 0.05, vs. empty or non-siRNA.P-499, P19CL6 cells stably transfected with pcDNA3.1-miR-499 recombinant plasmid; Empty, P-499 cells transfected with pcDNA3.1 plasmid; Sox6, P-499 cells transfected with pcDNA3.1-Sox6 recombinant plasmid.

We first determined whether miR-499 promoted proliferation through its target Sox6. P-499 cells were induced with DMSO for 8 days and then replated and transfected with pcDNA3.1-Sox6 (Sox6 overexpression plasmid) or pcDNA3.1 (empty) vector plasmid as a control. After another 2 days of culture, cell cycle analysis (Figure **6A** **, S4A**) and EdU incorporation assay were conducted (Figure **6B** **, S4B**). The results demonstrated that although miR-499 was overexpressed in these cells, the overexpression of Sox6 could inhibit the proliferation induced by miR-499, and the proliferation rate returned to normal.

Next, we performed an annexin V-FITC binding assay (Figure **6C** **, S4C**) in the P-499 cell line to determine whether miR-499 inhibited apoptosis through its target Sox6. Employing the same strategy described previously, after another 2 days of culture, we found that when Sox6 was overexpressed, the lower apoptosis rate induced by miR-499 was reversed to normal.

### MiR-499 might regulate cyclin D1 expression via its influence on Sox6

**Figure 7:**
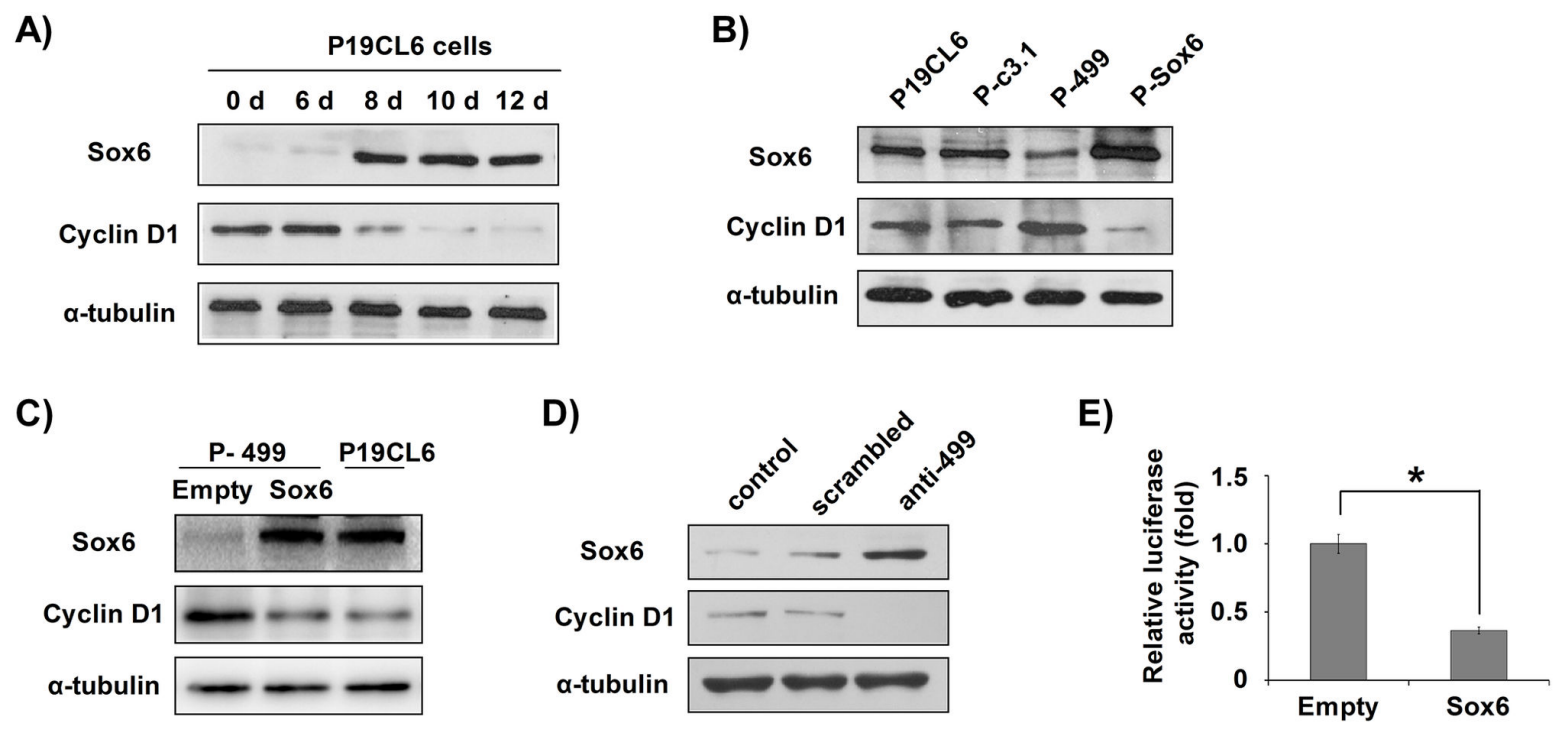
MiR-499 might regulate cyclin D1 expression via its influence on Sox6. (**A**) Inverse correlation between cyclin D1 and Sox6 in cardiac differentiation. Total cell lysates were collected at the indicated time points and analyzed by Western blotting with the corresponding antibodies as indicated. α-Tubulin was used as an internal control. (**B**) The cyclin D1 and Sox6 protein levels of differentiating cardiomyocytes on day 9 were compared between the different cell lines as indicated. (**C**) The Sox6 and cyclin D1 protein levels of P-499 cells overexpressed Sox6 were compared with the wild type P19CL6 cells. (**D**) Western blotting analysis was performed to detect Sox6, cyclin D1 in scrambled- and anti-499-transfected cells. (**E**) Luciferase reporter assay was used to detect the activity of the cyclin D1 promoter in the presence of Sox6 overexpression. The data are representative of three independent experiments that were repeated three times; each bar represents mean ± S.D. * *P* < 0.05, vs. vector.P-c3.1, P19CL6 cells stably transfected with pcDNA3.1 plasmid; P-499, P19CL6 cells stably transfected with pcDNA3.1-miR-499 recombinant plasmid; P-Sox6, P19CL6 cells stably transfected with pcDNA3.1-Sox6 recombinant plasmid. Empty, P-499 cells transfected with pcDNA3.1 plasmid; Sox6, P-499 cells transfected with pcDNA3.1-Sox6 recombinant plasmid.

We analyzed the expression profiles of Sox6 and cyclin D1 in P19CL6 cell lines by Western blotting analysis. In P19CL6 cells, from day 0 to day 6, when Sox6 was not expressed, the level of cyclin D1 protein was high; when Sox6 expression increased from day 8 to day 12, the expression of cyclin D1 was gradually attenuated, suggesting that increasing endogenous Sox6 expression is correlated with decreasing cyclin D1 expression (Figure **7A**).

To confirm the influence of Sox6 on cyclin D1, we compared the expression of Sox6 and cyclin D1 at the same time point – day 9 of differentiation. The results showed that when Sox6 was overexpressed in P-Sox6 cells, cyclin D1 expression was inhibited, whereas when Sox6 expression was inhibited in P-499 cells, cyclin D1 expression was enhanced (Figure **7B**). While, if we overexpressed Sox6 in P-499 cells, the enhanced expression of Cyclin D1 was significantly reduced to normal (Figure **7C**). These results indicate that expression of cyclin D1 is indeed downregulated by Sox6. Thus, the action of Sox6 as a cyclin D1 inhibitor could promote the exit of cardiomyocytes from the cell cycle during the late stage of cardiomyocyte differentiation. In addition, the effects of anti-499 were further examined by Western blotting analysis: miR-499 knock-down resulted in increased Sox6 expression, thus reduced cyclin D1 expression (Figure **7D**).

The 1,000-bp upstream region of the cyclin D1 promoter was cloned to create the pGL3-luciferase plasmid and was used with Sox6 overexpression plasmid to co-transfect P19CL6 cells on day 9. The results of the luciferase assay demonstrated that overexpression of Sox6 resulted in decreased luciferase activity of the cyclin D1 promoter. This indicates that Sox6 could negatively regulate transcription of the cyclin D1 gene (Figure **7E**).

## Discussion

The main aim of our study was to determine the association between Sox6 and miR-499 and its role during the process of cardiomyocyte differentiation and maturation.

As three highly conserved predicted miR-499-binding sites are reportedly present in Sox6-3’UTR and because of the known association between Sox6 and heart development, Sox6 is the most likely target gene of miR-499. This has been shown by luciferase reporter assays in various types of cell including 293T cells [24], 3T3 fibroblasts [7], COS cells [19] and HeLa cells (used in our study). Transgenic expression of miR-499 also effectively reduced the elevated Sox6 mRNA level in miR-208a^-/-^ hearts and reduced the Sox6 mRNA level in skeletal muscles of MCK-miR-499 transgenic mice [19]. In addition, Sox6 mRNA levels were significantly reduced in neonatal rat cardiomyocytes after miR-499 transfection [17]. In our experiments, not only did the endogenous Sox6 protein level decrease as a result of pre-miR-499 transfection, but also anti-499 transfection could upregulate the Sox6 protein level in P19CL6 cell-derived cardiomyocytes during the late stage of differentiation. This supports already available evidence that Sox6 is a target of miR-499.

As miRNAs usually show pronounced spatial and temporal expression patterns, we analyzed the time course of miR-499 expression and the expression of its target gene Sox6 during cardiomyocyte differentiation in the P19CL6 *in vitro* differentiation system. The early stage witnessed steady proliferation even as the cells began to differentiate; the expression of miR-499 and Sox6 was very low or undetectable at this stage. However, miR-499 and Sox6 were both highly expressed in the late stage, which is characterized by gradual decrease in proliferation. This indicates that the regulation of Sox6 by miR-499 is not only associated with cardiomyocyte differentiation but is also late stage-specific. In agreement with this, several studies have also reported that miR-499 is highly expressed in differentiated or post-mitotic cardiomyocytes but is almost absent or barely detectable in undifferentiated hCSCs [7], human cardiomyocyte progenitor cells (hCMPCs) [16] and hESCs [9]. However, how miR-499 is turned on in the cardiac differentiation system is still unclear.

As a potential target of miR-499, the expression of Sox6 is also late stage-specific. This has been supported by some studies although there are some controversial reports. Sluijter et al. [16] reported that Sox6 is expressed in proliferating hCMPCs. Hosoda et al. [7] reported that Sox6 mRNA expression was higher in the human myocardium than in hCSCs, while Sox6 proteins were barely detectable in human and rat myocytes but were apparent in both hCSCs and rat cardiac stem cells (rCSCs). In addition, in the P19CL6 *in vitro* differentiation system, our data showed that both Sox6 mRNA and Sox6 protein were highly expressed in the late stage of differentiation, which is consistent with the results of Cohen-Barak’s study [15].

It has been reported that transgenic mice expressing a high level of miR-499 had larger hearts and displayed contractile dysfunction. Moreover, under cardiac pressure overload by thoracic aortic banding, the hearts of miR-499 transgenic mice demonstrated accentuated cardiac enlargement and severe contractile dysfunction, but the cardiomyocyte size was almost normal [19]. Sox6 has also been reported to play a significant role in cardiogenesis. The mouse with *p* ^100H^ / *p* ^100H^ mutant, a Sox6 null mutant, is characterized by early postnatal lethality, associated with progressive atrioventricular heart block and myopathy [11].

It should be noted that the endogenous miR-499 and Sox6 showed the opposite expression trend during the cardiac differentiation of P19CL6 cells. Furthermore, when we knockdown the endogenous miR-499, the expression of Sox6 was increased. Meanwhile, miR-499 knock-down in P19CL6 cells had similar effects to Sox6 overexpression. Sox6 overexpression inhibited cell proliferation, which means that it might be required for the terminal differentiation of cells; at the same time, overexpression of Sox6 resulted in increased cell apoptosis, which was also observed in the case of miR-499 knock-down. Considering the observation in Sox6 null *p* ^100H^ mice [11], we presume that down-regulation of Sox6 expression not only prevents cardiomyocytes from withdrawing from the cell cycle, but also prevents them from undergoing maturation. These results indicate that the appropriate expression of Sox6 could facilitate cell cycle exit in cardiomyocytes and promote cardiomyocyte maturation concurrently with terminal differentiation. Thus, a balance between cell proliferation and apoptosis is required, and miR-499 probably plays a role in this. This is supported by our finding that overexpression of Sox6 reversed the enhanced proliferation and anti-apoptotic effects of miR-499.

Down-regulation of Cyclin D1 is required by cardiomyocytes for cell cycle exit and maturation during the late stage of differentiation. It has been reported that Sox6 inhibits cyclin D1 expression by interacting with β-catenin and HDAC1 in insulinoma INS-1E and NIH-3T3 cells [25] and that down-regulation of cyclin D1 is important for cell cycle exit [26]. In the early stage of differentiation, cyclin D1 might be regulated by some other mechanisms, rather than Sox6 (data not shown); however, in the late stage, the downregulation of cyclin D1 is important for cell cycle exit. In our study too, in P-Sox6 cells, overexpression of Sox6 reduced the level of cyclin D1 and may have caused premature growth arrest. In P-499 cells, as Sox6 levels were markedly decreased by overexpression of miR-499, cyclin D1 was consequently maintained at a higher level than in P19CL6 cells, leading to continuous proliferation of differentiating cardiomyocytes during the late stage of differentiation. These results indicate that miR-499 may target cyclin D1 via Sox6.

We have noticed some controversial reports on the function of miR-499. Two groups [7,16] have documented that miR-499 overexpression reduces proliferation and enhances differentiation of cardiac stem cells or cardiomyocyte progenitor cells; it increases the expression of cardiac troponin T, α-cardiac actinin, and MLC-2v or the expression of Nkx-2.5 and GATA4. In contrast, the data from our study showed that miR-499 overexpression has no remarkable effect on the expression of GATA4 and Nkx-2.5; moreover, the cell proliferation was significantly enhanced in P-499 cells. We think that the discrepancy in the findings is most probably related to differences in cell sources or types (mouse P19CL6 cells, human cardiac stem cells or human cardiomyocyte progenitor cells).

In conclusion, our results provide evidence for Sox6 being a target of miR-499, and for the role of both Sox6 and miR-499 in neonatal heart development. There are also some indications that miR-499 may target cyclin D1 via Sox6.

## Supporting Information

Figure S1 **Morphology of cells in different cell lines.** (**A**) Representative images show the morphology of P19CL6, P-c3.1, P-499 and P-Sox6 cells after DMSO induction for 6 days (upper panel) or 12 days (lower panel). (**B**) Representative images show the morphology of replated P19CL6, P-c3.1, P-499 and P-Sox6 cells. The cells were replated at day 10 of differentiation and cultured for another 2 days. (**C**) Representative images show the morphology of P19CL6 cells transfected with control, scrambled or anti-499 nucleotides at day 12 of differentiation and then cultured for another two days. P-c3.1, P19CL6 cells stably transfected with pcDNA3.1 plasmid; P-499, P19CL6 cells stably transfected with pcDNA3.1-miR-499 recombinant plasmid; P-Sox6, P19CL6 cells stably transfected with pcDNA3.1-Sox6 recombinant plasmid.
(TIF)

Figure S2 **MiR-499 participated in cell proliferation and apoptosis.** (**A**) Cell cycle analysis was performed by flow cytometry. Representative images of P19CL6, P-c3.1 and P-499 cells are shown. (**B**) EdU incorporation assay was performed. Representative images of P19CL6, P-c3.1 and P-499 cells are shown. (**C**) Cell apoptosis was analyzed with annexin V-FITC and PI staining by flow cytometry at the indicated time. Representative images of P19CL6, P-c3.1 and P-499 cells are shown. (**D**) The cells were cultured for 12 days after replating, and then analyzed with annexin V-FITC and PI staining by flow cytometry for apoptosis. Representative images of P19CL6 cells transfected with control scrambled or anti-499 nucleotides are shown. (**E**, **F**) The expression of miR-499 and Sox6 on day 4 after 1% DMSO induction was examined by real-time PCR and Western blotting respectively. GAPDH was used as an internal control. The experiment was repeated three times. Each bar represents mean ± S.D. * *P* < 0.05, vs. P-c3.1 cells. (**G**, **H**) The expression of miR-499 and Sox6 on day 11 after 1% DMSO induction was examined by real-time PCR and Western blotting respectively. GAPDH was used as an internal control. The experiment was repeated three times. Each bar represents mean ± S.D. * *P* < 0.05, vs. P-c3.1 cells. P-c3.1, P19CL6 cells stably transfected with pcDNA3.1 plasmid; P-499, P19CL6 cells stably transfected with pcDNA3.1-miR-499 recombinant plasmid; P-Sox6, P19CL6 cells stably transfected with pcDNA3.1-Sox6 recombinant plasmid.
(TIF)

Figure S3 **Sox6 participated in cell proliferation and apoptosis.** (**A**) Cell cycle analysis was performed by flow cytometry. Representative images of P19CL6, P-c3.1 and P-Sox6 cells are shown. (**B**) EdU incorporation assay was performed. Representative images of P19CL6, P-c3.1 and P-Sox6 cells are shown. (**C**) Cell apoptosis was analyzed with annexin V-FITC and PI staining by flow cytometry at the indicated times. Representative images of P19CL6, P-c3.1 and P-Sox6 cells are shown. P-c3.1, P19CL6 cells stably transfected with pcDNA3.1 plasmid; P-Sox6, P19CL6 cells stably transfected with pcDNA3.1-Sox6 recombinant plasmid.
(TIF)

Figure S4 **Sox6 reversed the proliferation and anti-apoptosis effects of miR-499.** (**A**) Cell cycle analysis was performed by flow cytometry. Representative images are shown. (**B**) EdU incorporation assay was performed. Representative images are shown. (**C**) Cell apoptosis was analyzed with annexin V-FITC and PI staining by flow cytometry at the indicated times. Representative images are shown. P-499, P19CL6 cells stably transfected with pcDNA3.1-miR-499 recombinant plasmid; Empty, P-499 or mir-499 cells transfected with pcDNA3.1 plasmid; Sox6, P-499 or mir-499 cells transfected with pcDNA3.1-Sox6 recombinant plasmid.
(TIF)

Video S1 (WMV)
