# Supplementary figures and images for: MiR-499 Regulates Cell Proliferation and Apoptosis during Late-Stage Cardiac Differentiation via Sox6 and Cyclin D1
Source: PLoS One. 2013 Sep 11;8(9):e74504. doi: 10.1371/journal.pone.0074504 (PMC3770584; doi:10.1371/journal.pone.0074504)

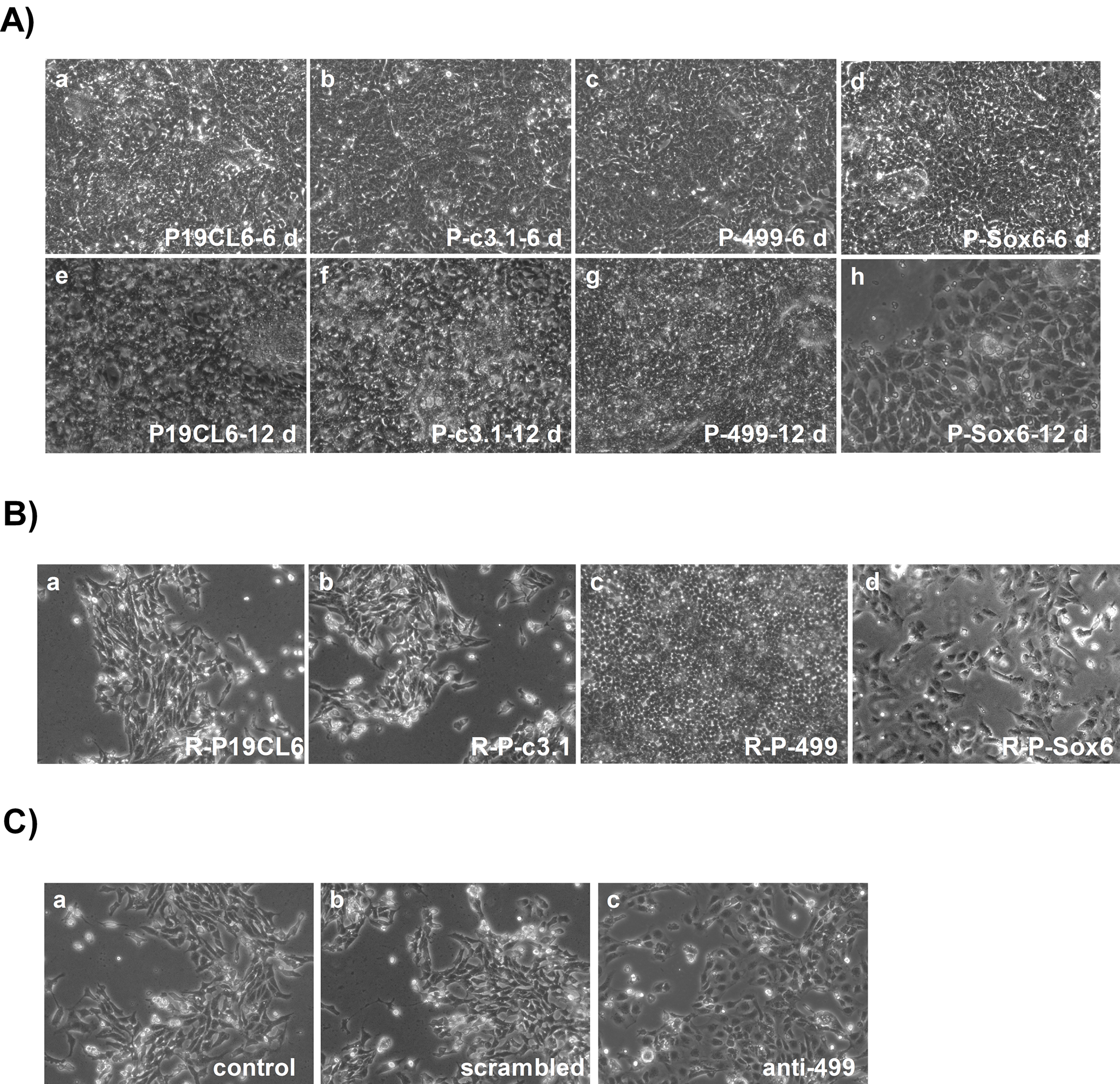

Supplement: Figure S1 — Morphology of cells in different cell lines. (A) Representative images show the morphology of P19CL6, P-c3.1, P-499 and P-Sox6 cells after DMSO induction for 6 days (upper panel) or 12 days (lower panel). (B) Representative images show the morphology of replated P19CL6, P-c3.1, P-499 and P-Sox6 cells. The cells were replated at day 10 of differentiation and cultured for another 2 days. (C) Representative images show the morphology of P19CL6 cells transfected with control, scrambled or anti-499 nucleotides at day 12 of differentiation and then cultured for another two days. P-c3.1, P19CL6 cells stably transfected with pcDNA3.1 plasmid; P-499, P19CL6 cells stably transfected with pcDNA3.1-miR-499 recombinant plasmid; P-Sox6, P19CL6 cells stably transfected with pcDNA3.1-Sox6 recombinant plasmid. (TIF) [file pone.0074504.s001.tif]

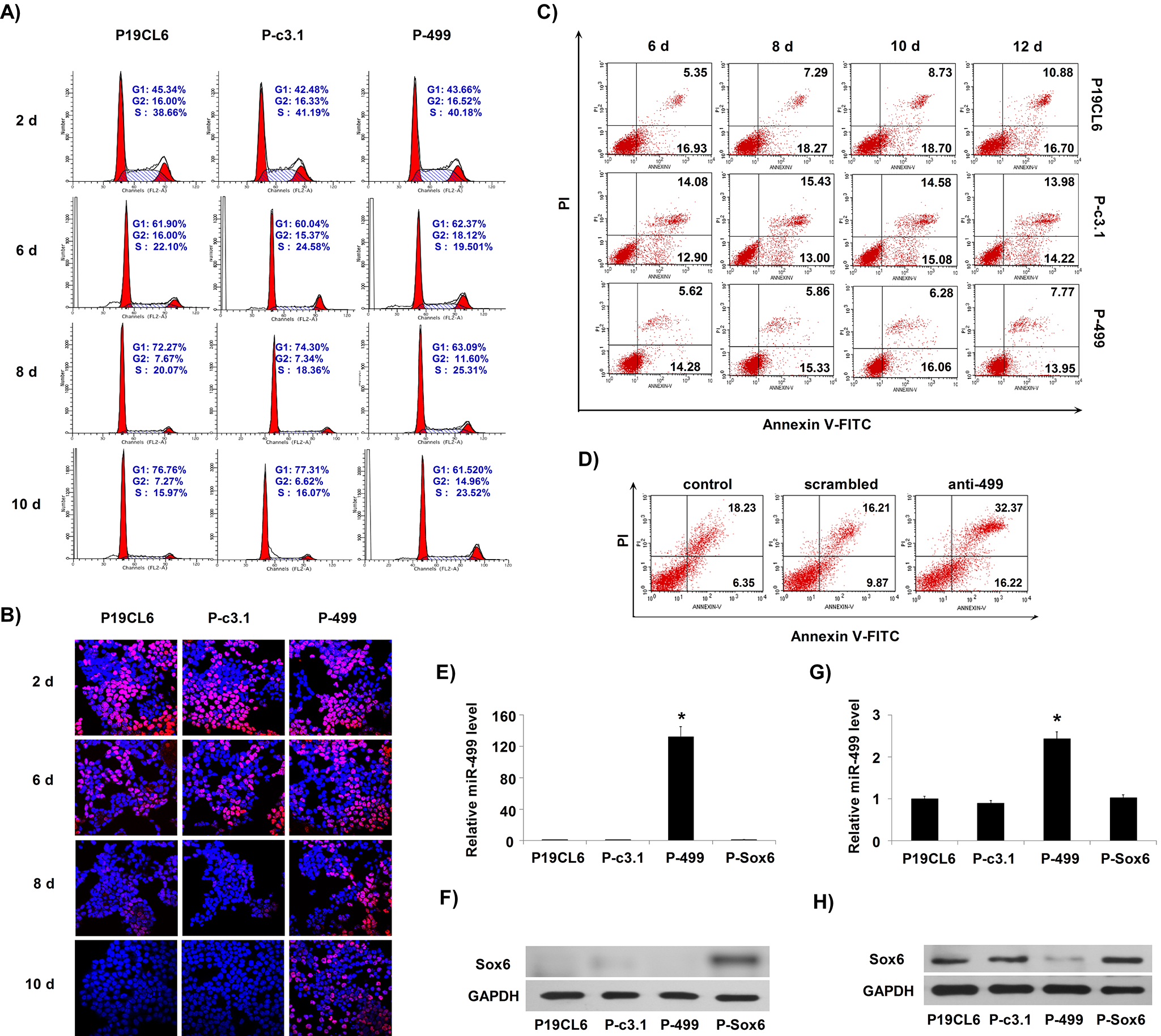

Supplement: Figure S2 — MiR-499 participated in cell proliferation and apoptosis. (A) Cell cycle analysis was performed by flow cytometry. Representative images of P19CL6, P-c3.1 and P-499 cells are shown. (B) EdU incorporation assay was performed. Representative images of P19CL6, P-c3.1 and P-499 cells are shown. (C) Cell apoptosis was analyzed with annexin V-FITC and PI staining by flow cytometry at the indicated time. Representative images of P19CL6, P-c3.1 and P-499 cells are shown. (D) The cells were cultured for 12 days after replating, and then analyzed with annexin V-FITC and PI staining by flow cytometry for apoptosis. Representative images of P19CL6 cells transfected with control scrambled or anti-499 nucleotides are shown. (E, F) The expression of miR-499 and Sox6 on day 4 after 1% DMSO induction was examined by real-time PCR and Western blotting respectively. GAPDH was used as an internal control. The experiment was repeated three times. Each bar represents mean ± S.D. * P < 0.05, vs. P-c3.1 cells. (G, H) The expression of miR-499 and Sox6 on day 11 after 1% DMSO induction was examined by real-time PCR and Western blotting respectively. GAPDH was used as an internal control. The experiment was repeated three times. Each bar represents mean ± S.D. * P < 0.05, vs. P-c3.1 cells. P-c3.1, P19CL6 cells stably transfected with pcDNA3.1 plasmid; P-499, P19CL6 cells stably transfected with pcDNA3.1-miR-499 recombinant plasmid; P-Sox6, P19CL6 cells stably transfected with pcDNA3.1-Sox6 recombinant plasmid. (TIF) [file pone.0074504.s002.tif]

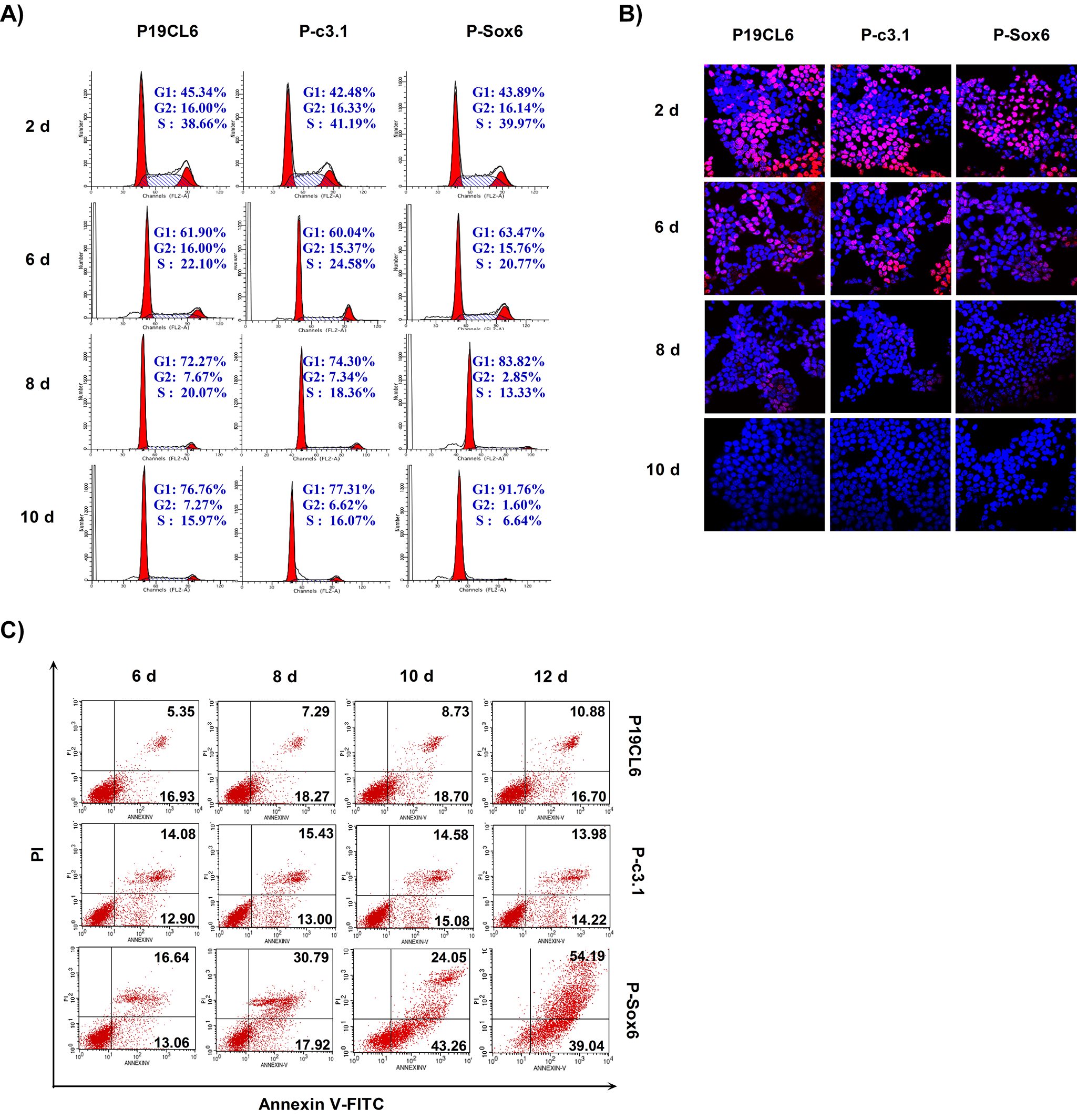

Supplement: Figure S3 — Sox6 participated in cell proliferation and apoptosis. (A) Cell cycle analysis was performed by flow cytometry. Representative images of P19CL6, P-c3.1 and P-Sox6 cells are shown. (B) EdU incorporation assay was performed. Representative images of P19CL6, P-c3.1 and P-Sox6 cells are shown. (C) Cell apoptosis was analyzed with annexin V-FITC and PI staining by flow cytometry at the indicated times. Representative images of P19CL6, P-c3.1 and P-Sox6 cells are shown. P-c3.1, P19CL6 cells stably transfected with pcDNA3.1 plasmid; P-Sox6, P19CL6 cells stably transfected with pcDNA3.1-Sox6 recombinant plasmid. (TIF) [file pone.0074504.s003.tif]

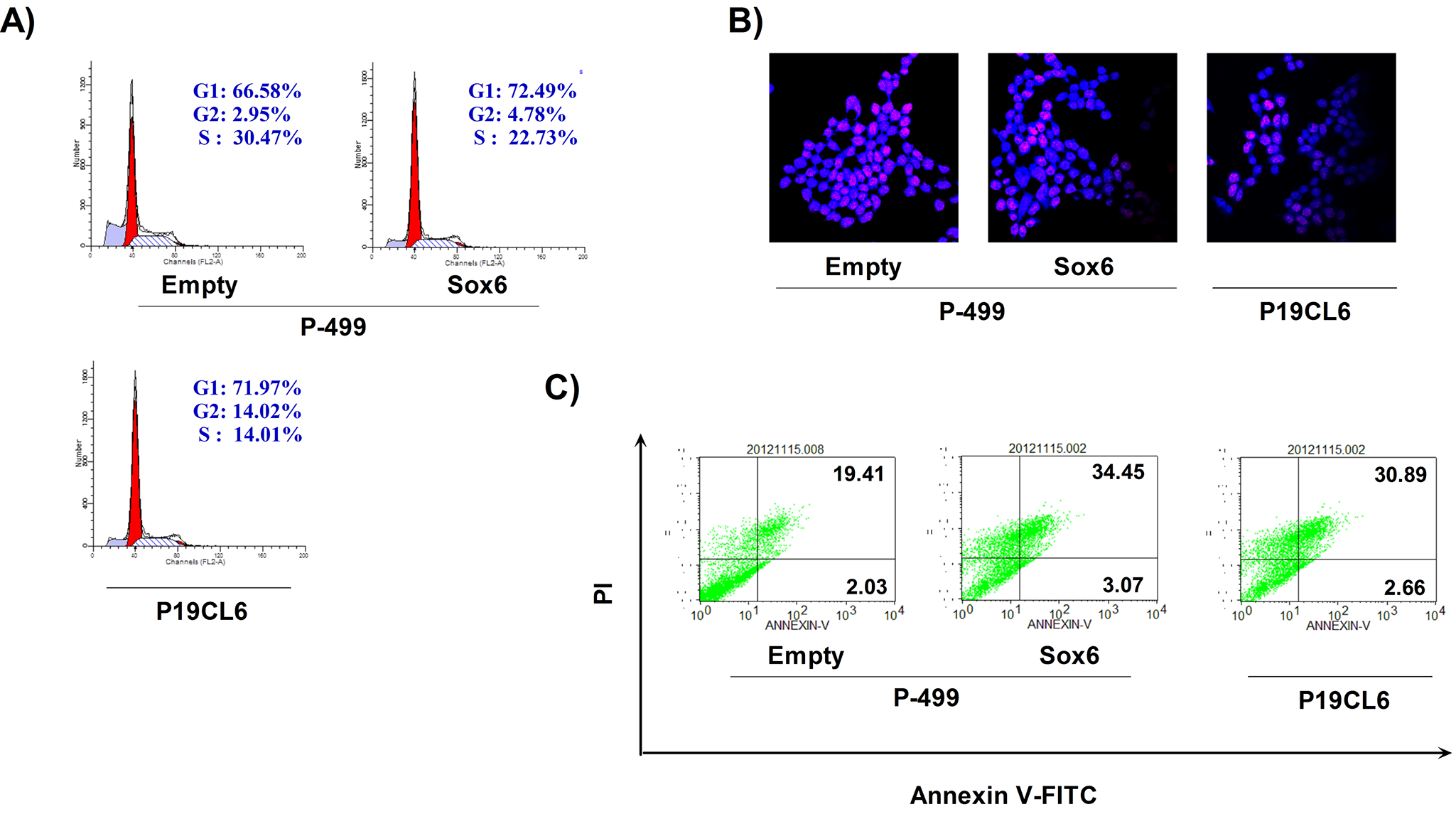

Supplement: Figure S4 — Sox6 reversed the proliferation and anti-apoptosis effects of miR-499. (A) Cell cycle analysis was performed by flow cytometry. Representative images are shown. (B) EdU incorporation assay was performed. Representative images are shown. (C) Cell apoptosis was analyzed with annexin V-FITC and PI staining by flow cytometry at the indicated times. Representative images are shown. P-499, P19CL6 cells stably transfected with pcDNA3.1-miR-499 recombinant plasmid; Empty, P-499 or mir-499 cells transfected with pcDNA3.1 plasmid; Sox6, P-499 or mir-499 cells transfected with pcDNA3.1-Sox6 recombinant plasmid. (TIF) [file pone.0074504.s004.tif]
